# Protease Enzyme Supplementation in Weaning Piglets Fed Reduced Crude Protein Diets: Effects on Gut Health Integrity and Performance Response

**DOI:** 10.3390/ani15142109

**Published:** 2025-07-17

**Authors:** Nathana Rudio Furlani, Stephane Alverina Briguente Da Motta, Bruno Teixeira Ramos, Wender Vieira Fernandes, Maria Rogervânia Silva de Farias, Rony Riveros, Tarciso Tizziani, Melissa Izabel Hannas

**Affiliations:** 1 Departament of Animal Science, Universidade Federal de Viçosa, Viçosa 36570-900, MG, Brazil; nathana.furlani@ufv.br (N.R.F.); stephane.motta@ufv.br (S.A.B.D.M.); bruno.t.ramos@ufv.br (B.T.R.); wender.fernandes@ufv.br (W.V.F.);; 2 Department of Animal Science, Sao Paulo State University, Jaboticabal 14884-900, SP, Brazil; rony.riveros@unesp.br; 3 SUNTAQ do Brasil, Jundiaí 13208-703, SP, Brazil; tarcisotizziani@additivanutrition.com

**Keywords:** digestibility, enzyme, protease, protein, swine

## Abstract

Two trials evaluated the effects of dietary protease inclusion in weaned piglets fed diets with or without crude protein (CP) reduction, focusing on performance, intestinal health, and amino acid digestibility. In Trial I, 270 piglets (21–63 days) received six treatments: control (PC), PC with 100 g/ton protease A (PC+A), CP reduced by 1.0% (NC1) or 1.5% (NC1.5), NC1.5 with 50 g/ton protease A (NC1.5+A), and NC1.5 with 50 g/ton protease B (NC1.5+B). PC+A improved weight gain, feed intake, and feed conversion compared with NC1.5+A. The incidence of diarrhea was reduced in animals fed protease-supplemented diets (PC+A, NC1.5+A and NC1.5+B). PC had greater ileal villus height than NC1.5+B, and PC+A showed a higher jejunal villus-to-crypt ratio than reduced CP groups. NC1.5+B increased jejunal expression of IL-6, TNF-α, and haptoglobin. In Trial II, 12 ileal-cannulated piglets received diets with or without protease A. Protease improved the standardized ileal digestibility (SID) of methionine+cysteine and tryptophan but reduced the SID of glycine and proline. While protease supplementation can improve some amino acids (Met+Cys and Thr) protein digestibility, our findings suggest it cannot fully replace careful amino acid balancing in CP-reduced diets. However, protease-supplemented diets were associated with improved intestinal morphometry and a reduced incidence of diarrhea.

## Simple Summary

Weaning is a critical stage in pig production that challenges piglets’ health and growth due to abrupt dietary and environmental changes. These stressors often impair gut development, reduce enzyme production, and disrupt the intestinal microbiota, increasing the risk of diarrhea and poor performance. High-protein diets commonly used after weaning can worsen these effects, particularly due to anti-nutritional factors in soybean meals. Supplementing piglet diets with exogenous proteases has been proposed as a strategy to improve protein digestibility and gut health. In this study, we evaluated whether adding two commercial proteases to reduced-protein diets could alleviate post-weaning stress in piglets. We examined effects on intestinal integrity, inflammation, diarrhea incidence, and growth, as well as nutrient digestibility. Our findings contribute to understanding how dietary enzyme supplementation may support healthier and more efficient swine production during this vulnerable phase.

## 1. Introduction

The weaning phase is one of the most critical periods in swine production, with several implications for the long-term welfare and productive performance of piglets. During this stage, piglets are exposed to multiple stressors, including the abrupt transition from liquid to solid feed, psychosocial changes, and alterations in environmental conditions. These stressors collectively contribute to reduced feed intake, which in turn compromises the development of the gastrointestinal tract (GT) and the maturation of the immune system. Such disruptions can lead to an imbalance in gut microbiota establishment, triggering a cascade of adverse effects on piglet health and growth [1,2]. An underdeveloped GT results in diminished nutrient absorption and limited production and secretion of endogenous enzymes, further exacerbating the challenges faced during this phase [3].

The situation is often aggravated by the composition of post-weaning diets, which are typically high in crude protein (CP) to support early growth. Soybean meal (SBM), a common ingredient in these diets due to its high protein content, contains anti-nutritional factors (ANFs) that inhibit the action of endogenous proteases (e.g., pepsin, trypsin, and chymotrypsin). This inhibition is particularly problematic during the post-weaning period because the production of these enzymes declines significantly during the transition [4,5]. In this sense, undigested protein reaching the hindgut can increase protein fermentation, leading to the production of toxic metabolites and the proliferation of non-beneficial bacteria. This can compromise intestinal barrier function, induce inflammation, and elevate the risk of post-weaning diarrhea, ultimately impairing animal growth [6,7]. Thus, the combination of high-PC diets and an immature GT exacerbates the stress associated with weaning.

To address these challenges, exogenous protease supplementation has been routinely adopted in swine production. Proteases are enzymes that assist in protein degradation, thereby improving the digestibility of CP, energy, and amino acids at both the ileal and total tract levels [8,9,10]. By breaking down cell walls and glycosidic bonds, proteases degrade ANFs, such as protease inhibitors, antigenic proteins, and non-protein amino acids, which are resistant to endogenous enzyme activity. However, the efficacy of exogenous proteases remains controversial, particularly in diets with reduced CP content due to decreased SBM inclusion [11]. This divergence in results may stem from a limited understanding of the mechanisms underlying protease action and their interactions with dietary components [8,9].

We hypothesized that supplementing CP-reduced diets with two exogenous commercial proteases during the post-weaning period could mitigate the adverse effects of post-weaning stressors. Protease supplementation may enhance gut health, improve nutrient absorption, and prevent performance declines in piglets. This study aims to evaluate the impact of protease inclusion in CP-reduced diets on gut integrity, inflammatory markers, and diarrhea incidence, as well as its capacity to improve growth performance. Additionally, a standard digestibility trial was conducted in barrows to assess the digestibility of essential and non-essential amino acids.

## 2. Materials and Methods

**Trial I**

The protocol for this experiment was reviewed and approved by the Ethics Committee for the Use of Production Animals at the Universidade Federal de Viçosa (protocol number 80/2023), and it adhered to the rules of the National Council for the Control of Animal Experimentation. The experiment was conducted at a commercial farm in Fazenda Vitória, Santo Antônio do Grama, Minas Gerais, Brazil.

### 2.1. Experimental Design, Animals, and Diets

A total of 270 female and non-castrated male piglets were used in this study. The animals were crossbred pigs from DB90 × AGPIC 337 (DB Agricultura e Pecuária and Agroceres PIC, Patos de Minas, MG, Brazil). Piglets were weaned at 21 days of age, with an average body weight of 5.30 ± 0.65 kg. The animals were assigned to six treatments in a randomized block design. Each treatment consisted of nine replicates, with five animals per pen, considering the pen as the experimental unit. Blocking criteria were based on initial body weight.

The dietary treatments included: PC: a positive control diet containing 21.78% crude protein (CP) with adequate levels of essential amino acids (AAs); PC+A: the PC diet supplemented with 100 g/ton of commercial protease A; NC1: a negative control diet with a 1.0% reduction in CP and proportionally reduced essential AAs to maintain the same AA-to-PC ratio; NC1.5: a negative control diet with a 1.5% reduction in CP, and proportionally reduced essential AAs; NC1.5+A: the NC1.5 diet supplemented with 50 g/ton of commercial protease A; NC1.5+B: the NC1.5 diet supplemented with 50 g/ton of commercial protease B. Protease A, a neutral–alkaline protease derived from *Aspergillus* spp. and *Bacillus* spp., which are of fungal and microbial origins and are adapted to function across a wide pH range from 7 to 11. Protease B, an alkaline protease produced by *Bacillus licheniformis*, an organism that acts at a pH of 9–11.

The feeding program consisted of three phases: weaner (21–32 days), grower (32–42 days), and finisher (42–63 days). Diets (Table 1) were formulated according to the recommendations of Rostagno et al. [12]. The basal diet contained corn and soybean meal, supplemented with minerals, vitamins, and phytase at 500 FTU/kg, representing a practical diet. The nutritional composition of the experimental diets analyzed is shown in the Supplementary Material (Table S1).

Piglets were housed in group pens (1.75 × 1.00 m) equipped with semi-automatic feeders and nipple drinkers, located in a masonry barn with a concrete floor, ceramic tile roof, and side curtains. Animals had ad libitum access to water and feed throughout the trial.

### 2.2. Performance Response

Body weight (BW) was recorded at 21, 32, 42, and 63 days old, corresponding to the transition of feeding phases. Feed intake was measured at the same interval. From these data, the average daily weight gain (ADG), average daily feed intake (ADFI), and feed conversion ratio (FCR) were calculated.

### 2.3. Incidence of Diarrhea

During the first seven days of each phase (21–28 days, 32–39 days, and 42–49 days), fecal consistency was in situ visually assessed using the scale described by [13]. Fresh feces were scored according to the following scale: 1 = solid; 2 = semi-solid; 3 = semi-liquid; and 4 = liquid. The evaluation was conducted as a single-blind procedure, where the evaluator did not have access to the treatment description per pen.

Diarrhea incidence (DI) was defined as the production of feces scored as 3 or 4 for two consecutive days.

$$DI\left(\%\right)=\frac{\mathrm{numbler}\mathrm{of}\mathrm{pigs}\mathrm{with}\mathrm{diarrhea}\mathrm{in}\mathrm{each}\mathrm{pen}\times \mathrm{diarrhea}\mathrm{days}}{\mathrm{numbler}\mathrm{of}\mathrm{piglets}\times 7\mathrm{days}\mathrm{of}\mathrm{evaluation}}\times 100$$

### 2.4. Intestinal Tissue

At 32 days of age, two piglets with a BW closest to the pen were selected for euthanasia, electrically stunned, followed by exsanguination to collect intestinal samplings. Segments of the duodenum, jejunum, and ileum were collected and then washed in physiological solution and fixed in 4.0% paraformaldehyde solution for 24 h at room temperature for morphometric analysis.

To collect jejunum samples for gene expression analysis, all instruments that came into contact with the tissue were sanitized with 70% alcohol, dried with a paper towel, and sprayed with RNAse Exterminator (Protech Technology Enterprise Co., Ltd., Nanking Dist., Taipei, Taiwan) between each sampling. The portion of the sampled jejunum was washed with sterile saline solution to remove the digesta in contact with the tissue. From the tissue sample, about ten 0.25 cm^3^ subsamples were collected, washed individually in sterile saline solution serum, placed in cryotubes with a capacity of 1.8 mL, frozen instantaneously in liquid nitrogen, and stored at −80 °C for further analysis.

### 2.5. Morphometric Evaluation

Intestinal samples were segments of 2.0 × 2.0 cm of the intestine, corresponding to the duodenum (10 cm from the pylorus), the jejunum (the middle portion), and the ileum (5 cm proximal to the ileocecal junction), according to [14]. The tissue was washed and fixed in 4.0% paraformaldehyde solution (24 h). The samples were cut transversely, washed in ethanol gradients, clarified in xylene, and embedded in liquid paraffin at 65 °C. Three transverse cuts with a thickness of 5 μm each were placed on each slide and stained with hematoxylin and eosin (H&E). Villus height (VH) and crypt depth (CD) were measured using EVOS M5000 Imaging System (Invitrogen, Carlsbad, CA, USA; Thermo Fisher Scientific, Beverly, CA, USA) microscope at 10× magnification. The images were analyzed using the ImageJ 1.50i software (National Institutes of Health, Bethesda, MD, USA). A quantity of 20 villi and their 20 crypts were selected and measured. Villus:crypt ratios using the length data were the calculated. A single individual made all measurements.

### 2.6. Gene Expression of Pro-Inflammatory Proteins

Total RNA was extracted from the jejunum samples using Trizol^®^ (Invitrogen, Carlsbad, CA, USA) and purified with the PureLink™ RNA Mini Kit (Invitrogen, Carlsbad, CA, USA). RNA concentration was measured with a NanoDrop™ Lite spectrophotometer (Thermo Fisher Scientific, Beverly, CA, USA), and integrity was verified by 1.0% agarose gel electrophoresis. cDNA synthesis was performed using the High-Capacity cDNA Reverse Transcription Kit (Applied Biosystems, Thermo Fisher Scientific, Beverly, CA, USA). qRT-PCR was conducted in duplicate on a QuantStudio 3 thermocycler (Applied Biosystem, Foster City, CA, USA) with SYBR^®^ Green (Applied Biosystems, Foster City, CA, USA) and GoTaq^®^ qPCR Master Mix (Promega, Madison, WI, USA). The cycling protocol included an initial denaturation at 95 °C for 2 min, followed by 40 cycles of denaturation (95 °C, 15 s) and annealing and extension at 60 °C for 1 min. A standard curve (R^2^ ≥ 0.97) was generated from six cDNA dilutions for mRNA quantification [15].

The target genes included pro-inflammatory cytokines (tumor necrosis factor-alpha—TNF-α, interleukin 1 beta—IL-1β, interleukin 6—IL-6, and haptoglobin), anti-inflammatory cytokines (interleukin 10—IL-10 and transforming growth factor beta 1—TGF-β1), junctional proteins (occluding—OCL and zonula occludens-1—ZO-1), and a genetic normalizer (18S). The primer sequences for qRT-PCR were obtained from GenBank (Table 2).

**Trial II**

This research was carried out at the Teaching, Research and Extension Unit in Swine Production and Nutrition of the Department of Animal Science of the Universidade Federal de Viçosa, Viçosa, Minas Gerais, Brazil.

### 2.7. Surgery and Sample Collection

T-cannulas were implanted following a technique adapted from Donkoh et al. [16], positioning the cannula in the distal ileum, 20 cm from the ileocecal valve. Before surgery, pigs fasted for 12 h to reduce digesta contamination. After anesthesia induction, animals were placed in left lateral recumbency, and we performed extensive trichotomy and aseptic preparation of the surgical site. A 5–6 cm vertical incision was made in the flank region, 3–4 cm caudal vertebra to the last rib. We located and exteriorized the small intestine and then made a 2–2.5 cm incision along the antimesenteric side, 10–15 cm cranial to the ileocecal junction.

Following surgery, pigs recovered in pens with 2.3 m × 2.16 m × 0.95 m (length × width × height), equipped with individual dry feeders and bowl-type drinkers. The shed was previously sanitized with soap and water and later disinfected with quicklime, water, and disinfectant solution.

For five days post-surgery, they received therapeutic levels of antibiotics as a prophylactic measure. We reintroduced feed gradually on the first day and then provided it ad libitum. The recovery diet, based on corn and soybean meal, met all nutritional requirements, according to Rostagno et al. [12]. The skin suture was removed 15 days after surgery. The ileal digestibility test with a simple T-cannula was performed 15 days after surgery, considering the recovery of all animals.

### 2.8. Diets and Experimental Design

Twelve castrated male pigs (Agroceres PIC, Patos de minas, MG, Brazil, Camborough × AGPIC 412) with an initial average body weight of 34.67 ± 1.48 kg were distributed in an incomplete Latin square design, with three diets and two periods of 7 days in each square, totaling eight observations per treatment. In the second period, the animals were changed to a treatment to exclude the effects of the individual on digestibility values. The dietary treatments included three groups (Table 3): a basal diet with 30% soybean meal (SBM); a basal diet with 30% SBM and protease A supplementation (basal diet + protease A); and a purified nitrogen-free diet (N-free) used to determine protein and amino acid endogenous loss. Titanium dioxide (TiO_2_) was included in all diets at 0.5% as an indigestibility marker, and minerals and vitamins were included to meet or exceed the requirements for growing pigs, as recommended by Rostagno et al. [12].

### 2.9. Experimental Procedure

Pigs were fed twice daily (8 am and 5 pm) based on metabolic body weight (BW^0.75^ kg), with the diet mixed with water in a 1:1 ratio to minimize feed waste and ensure complete intake. Throughout the experiment, the animals had free access to water. Before data collection, the animals were subjected to a five-day adaptation period, followed by two consecutive days of ileal digesta collection over 10 continuous hours.

The ileal digesta was collected using plastic bags fixed directly to the cannula (5 cm × 20 cm), which were removed after filling and replaced by new plastic bags; the samples were immediately frozen at −20 °C to prevent amino acid degradation.

After the collection period, the animals rested for five days, receiving a diet based on corn and soybean meals to meet the nutritional requirements described by Rostagno et al. [12].

During the second collection period, one animal submitted to N-free treatment presented cannula rejection and was removed from the evaluation by a specialist.

### 2.10. Chemical Analysis

At the end of the experiment, the digesta samples were thawed, homogenized, lyophilized, and ground for each experimental unit and period. A subsample was collected for chemical analysis, totaling eight replicates per treatment.

Both the diet and the ileal digesta samples were analyzed for dry matter (DM), nitrogen (N), titanium dioxide (TiO_2_), and amino acid (AA) content using the following methods. The dry matter was determined using the INCT-CA G-003/1 method. Nitrogen content was determined by the Kjeldahl method. Titanium dioxide concentration was performed using the INCT-CA M-007/1 method, as described by [17] and performed at the Laboratory of Animal Nutrition of the Department of Animal Science of the Universidade Federal de Viçosa, Minas Gerais, Brazil; the amino acid content in the feed and digesta was determined by high pressure liquid chromatography (HPLC) at the CBO Laboratory (Valinhos, São Paulo, Brazil), according to the methods described by [18,19,20]. The crude protein was calculated using a conversion factor of 6.25 based on nitrogen content.

### 2.11. Calculation of Standardized Digestible Coefficients

The coefficients of standardized ileal digestibility (CSID) of CP and of AAs were calculated using a direct method. The following formulas were used to determine the CSID [21] and basal endogenous loss (BEL):

Where:

FI1 = Indigestibility factor of the test diet:

$$FI1=\frac{\%\mathrm{TiO2}\mathrm{Test}\mathrm{Diet}}{\%\mathrm{TiO2}\mathrm{Test}\mathrm{Digest}}$$

Coefficient of apparent ileal digestibility of crude protein (CAID CP%):

$$\mathrm{CAID}\mathrm{CP}\left(\%\right)=\frac{\left[\left(\%\mathrm{CP}\mathrm{diet}-\left(\%\mathrm{CP}\mathrm{digest}\times FI1\right)\right)\right]}{\%\mathrm{CP}\mathrm{diet}}\times 100$$

FI2 = Indigestibility factor of the protein-free diet (N-free diet).

Coefficient of standardized ileal digestibility of crude protein (CSID CP %):

$$\mathrm{CSID}\mathrm{CP}\left(\%\right)=\frac{\left[\left(\%\mathrm{CP}\mathrm{diet}-\left(\%\mathrm{CP}\mathrm{digest}\times FI1\right)-(\%\mathrm{CP}\mathrm{diet}\times FI2)\right)\right]}{\%\mathrm{CP}\mathrm{diet}}\times 100$$

where:

E1 = Digesta test diet.

Coefficient of apparent ileal digestibility of amino acids (CAID AAs):

$$\mathrm{CAID}\mathrm{AA}\left(\%\right)=\frac{\left[\left(\mathrm{mg}\mathrm{AA}/g\;\mathrm{diet}-\left(\mathrm{mg}\mathrm{AA}/g\;E1\times FI1\right)\right)\right]}{\mathrm{mg}\mathrm{AA}\mathrm{in}\mathrm{diet}}\times 100$$

E2 = Digesta N-free diet.

Coefficient of standardized ileal digestibility coefficient of amino acids (CSID AA%):

$$\mathrm{CSID}\mathrm{AA}\left(\%\right)=\frac{\left[\left(\mathrm{mg}\mathrm{AA}/g\;\mathrm{diet}-\left(\mathrm{mg}\mathrm{AA}/g\;E1\times FI1\right)-(\mathrm{mg}\mathrm{AA}/g\;E2\times FI2)\right)\right]}{\mathrm{mg}\mathrm{AA}\mathrm{in}\mathrm{diet}}\times 100$$

The following formula was used to determine basal ileal endogenous amino acid losses [22].

Ileal basal endogenous losses of amino acids (BELs):

BasalIlealAAend=mgAA/g digest×(%TiO2NFreediet/ %TiO2digest)

### 2.12. Statistical Analysis

All statistical analyses were performed using R software version 4.3.1 (RStudio, Boston, MA, USA, 2023). Pens were considered the experimental units for performance and diarrhea score analyses, while one piglet per pen was used for intestinal morphometry and mRNA expression analyses. Data were subjected to analysis of variance (ANOVA), and treatment means were compared using the Tukey test when significant differences were detected (*p* ≤ 0.05). The incidence of diarrhea and fecal scores were evaluated across different age groups (21–27 days, 32–38 days, and 42 to 48 days). Data analyses were performed by accounting for phases, treatment effects, and their interactions, with repeated measures (phases as longitudinal factors). When assessing the effects of the treatments and phases, the results were compared using the Duncan test at a 95% confidence level. Digestibility coefficients were calculated from replicate means and analyzed by ANOVA, with significance set at *p* < 0.05.

## 3. Results

### 3.1. Performance

The effects of different dietary treatments on growth performance and feed efficiency were evaluated across three phases (21–32, 32–42, and 42–63 days) as well as cumulatively (21–63 days) (Table 4).

During the first phase (21–32 days), two piglets from the PC group died, necessitating adjustments for the ADFI and the FCR. No significant differences were detected (*p* = 0.088), although numerical trends suggested a higher intake in the PC (0.306 g/d) and PC+A (0.310 g/d) diets. However, the ADG decreased (*p* < 0.05) in the reduced-crude protein groups supplemented with protease (NC1.5+A and NC1.5+B) compared with the PC and PC+A diets. Similarly, the FCR was significantly affected by treatment (*p* < 0.05), with the PC (1.362) and PC+A (1.369) diets showing an improved FCR compared with the NC1.5+A (1.591) diet.

In the second phase (32–42 days), the ADFI showed no significant effects (*p* = 0.314), although the NC1 (0.610 g/d) diet had a numerically higher intake. The ADG was higher in the PC+A diet than in the NC1.5+A and NC1.5+B (*p* < 0.05) diets, leading to a better FCR for the PC+A. Protease supplementation in the PC diet improved the ADG, with the NC1.5+A diet exhibiting a better FCR recovery than the NC1.5+B.

During the third phase (42–63 days), the PC+A diet had a higher ADFI than both NC1.5 groups with protease supplementation, while other treatments matched the PC diet. The PC+A and PC diets also achieved a higher ADG than the NC1.5+A (*p* < 0.05) diet, yielding an FCR similar to the NC1.5. Notably, the NC1 (mild CP reduction) diet did not affect performance, matching the NC1.5+B throughout the trial. Over the entire period (21–63 days), piglets that were fed regular CP diets of PC and PC+A also outperformed the NC1.5+B diet in both the ADG and the ADFI. However, the protease source B in the NC1.5+B diet failed to match the PC diet’s ADG. The FCR improved significantly in the PC+A diet over the NC1.5 and NC1.5+A (*p* < 0.05) diets.

### 3.2. Diarrhea Incidence and Fecal Score

**Figure 1:**
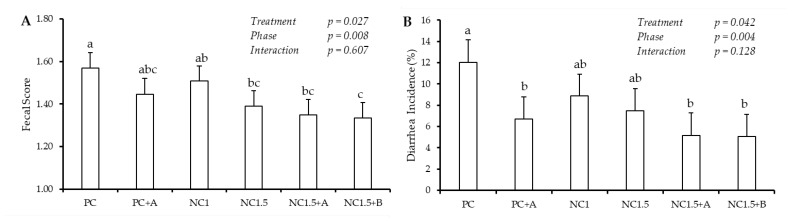
Effects of protease supplementation in diets with regular protein levels or reduced protein levels on fecal scores and incidence of diarrhea. PC: positive control diet; PC+A: PC diet supplemented with 100 g/ton of commercial protease A; NC1: negative control diet with a 1.0% reduction in crude protein (CP) and amino acids; NC1.5: negative control diet with a 1.5% reduction in CP and amino acids; NC1.5+A: NC1.5 diet supplemented with 50 g/ton of protease A; NC1.5+B: NC1.5 diet supplemented with 50 g/ton of protease B. (**A**) Fecal score and (**B**) diarrhea incidence presented a constant reduction with age (phases) increase. Since no interaction (treatment × phase) was observed for any response variables, only the treatment effect was plotted. a, b, c: Means in the row followed by distinct letters differ by the Duncan test (*p* < 0.05).

Figure 1 shows the effects of protease supplementation in diets with either regular or reduced protein levels on fecal scores and diarrhea incidence across different experimental phases. There were significant effects (*p* < 0.05) of the phases and treatments. However, no interaction between the phases and treatments was observed. Piglets that were fed a diet supplemented with protease (PC+A, NC1.5+A, and NC1.5+B) presented a reduction in diarrhea incidence, and the animals fed the NC1.5+B diet showed a lower fecal score than the PC group. There was a higher incidence of diarrhea in phases 1 and 2 and a lower fecal score in phase 2 (*p* < 0.05).

### 3.3. Gut Morphometry

Table 5 presents the intestinal morphometry variables across different segments of the small intestine. No significant differences (*p* > 0.05) occurred in any morphometric parameters among treatment groups for the duodenum segment. On the other hand, dietary treatments had a significant effect on jejunal morphology (*p* < 0.05). While villus height (VH) remained unchanged, crypt depth (CD) increased significantly in the NC1.5+A group compared with PC+A. Other treatments showed intermediate CD values that did not differ statistically from one another. Consequently, the PC+A group demonstrated a higher ratio of villus height to crypt depth (VH:CD) ratio than the reduced CP groups (NC1.5 and NC1.5+A). For the ileal segment, only VH differed among treatments, with piglets fed the PC diet showing greater VH values than those fed NC1.5+B (*p* < 0.05). No other morphometric parameters varied significantly between the treatment groups for this segment.

### 3.4. Immune Response

Gene expression analysis of the principal immune response proteins revealed distinct patterns among the studied proteins (Table 6). Pro-inflammatory proteins showed significant differential expression across the treatment groups. IL-6 expression levels were markedly higher in the NC1.5+B group compared with both the PC+A and the NC1 groups (*p* < 0.05). Similarly, TNF-α expression increased significantly in the NC1.5+B group relative to the PC, its protease-supplemented counterpart PC+A, and the NC1.5. Haptoglobin expression followed this trend, with the NC1.5+B group demonstrating the highest levels among all the treatments, followed by the NC1.5+A (*p* < 0.05). In contrast, IL-1β expression remained unaffected by the dietary treatments, although numerical trends suggested a slightly higher expression in the NC1.5+A and NC1 groups compared with the PC+A.

The anti-inflammatory proteins IL-10 and TGF-β1 showed no significant variation in expression across the treatment groups. Similarly, analysis of junction proteins ZO-1 and occludin revealed no statistically significant dietary effects (*p* > 0.05). However, ZO-1 expression showed a trend toward higher values in the NC1 and NC1.5+B groups compared with the PC and PC+A groups.

### 3.5. Amino Acid Digestibility

Analysis of CP and SID coefficients is shown in Table 7. While crude protein digestibility remained unaffected by protease supplementation, the essential amino acids Met+Cys and Trp showed significant enhancements (*p* < 0.05). The SID of Met+Cys improved from 87.79% to 94.52%, and Trp increased from 74.53% to 81.90% with protease addition. Interestingly, the non-essential amino acids glycine and proline exhibited higher SID values in the basal diet compared with the protease-supplemented diets (*p* < 0.05). Cysteine SID demonstrated the most pronounced improvement, rising from 82.74% to 93.52% with protease supplementation. The apparent ileal digestibility of amino acids follows the same pattern as SID coefficients (Table S2).

Baseline endogenous losses were determined by the nitrogen-free diet (g/kg of daily DM intake): lysine, 0.340; methionine, 0.122; threonine, 0.500; tryptophan, 0.223; arginine, 0.364; valine, 0.426; isoleucine, 0.311; leucine, 0.555; histidine, 0.172; phenylalanine, 0.314; alanine, 0.524; cysteine, 0.122; tyrosine, 0.232; glycine, 0.890; serine, 0.531; proline, 1.189; glutamic acid, 0.740; and aspartic acid, 0.416.

## 4. Discussion

Protein and amino acid deficiencies in swine diets impair growth and cause undernutrition, leading to weight loss, poor lean mass deposition, reduced feed efficiency, and increased production costs [23]. Conversely, excessive CP, particularly in diets based on SBM as the primary source of protein, during post-weaning diets may yield conflicting results due to undigested protein fermentation in the hindgut or inflammatory responses triggered by SBM’s anti-nutritional factors. Several studies demonstrate that protease supplementation in SBM-based diets improves performance in piglets fed CP-reduced diets [24]. Although the weaning transition represents a stressful period for piglets adapting to solid feed, reducing dietary CP does not appear to significantly impair performance. In the present study, we evaluated SBM-based diets with reduced CP and amino acid levels, hypothesizing that protease supplementation would compensate for essential amino acid reductions while maintaining performance and supporting gut health and immune function. An alternative approach worth investigating would involve CP reduction while maintaining essential amino acid levels, which can be evaluated further.

Our findings corroborate previous studies indicating that protease supplementation enhances feed efficiency but may limit maximal growth [25]. The improved FCR observed with a regular CP level (PC treatment) with protease supplementation aligns with reports of enhanced nutrient digestibility in enzyme-supplemented diets [26]. The absence of treatment effects on ADFI suggests that the 1% and 1.5% CP reduction still met the animals’ nutritional requirements, as supported by Munezero et al. [5], who reported no ADFI differences in pigs aged 15–63 days fed diets with a 0.5% CP reduction. Similar findings were reported with a 1.5% CP reduction [11,27].

While protease supplementation improved certain digestibility parameters on the digestibility trial in older pigs than that used in the post-weaning piglets, it failed to fully compensate for performance deficits in CP-reduced diets, particularly at the 1.5% reduction level. Diets with a 1% CP reduction maintained performance comparable to the positive control (PC). Throughout the 21–63 day period, piglets fed the PC diet and the PC supplemented with protease (PC+A) demonstrated a superior average daily gain (ADG) and FCR compared with the CP-reduced diets with protease supplementation (NC1.5+A and NC1.5+B). These results agree with Wang et al. [27], who found that exogenous protease failed to improve performance in amino acid-reduced diets throughout the wean-to-finish period. It is important to mention that many studies reduce a maximum of 1% of CP maintaining the same SID lysine to metabolizable energy ratio. The 1–1.5% CP reduction in our study may have been excessive, as the PC and PC+A diets—which met nutritional requirements and used higher protease doses (100 mg/kg)—yielded optimal performance. This observation was supported by the research of Yu et al. [24], which reported intermediate performance between control and protease-supplemented CP-reduced diets.

Protease supplementation in regular CP diets appeared to improve jejunal morphology, suggesting enhanced nutrient absorption [28]. The jejunal VH:CP ratio showed the most pronounced response to dietary treatments. Additionally, ileal VH differed significantly with protease supplementation, indicating segment-specific adaptation, consistent with Song et al. [29]. Protease A particularly enhanced gut structure, likely by modulating microbial metabolites (e.g., short-chain fatty acids) and reducing anti-nutritional factors in regular CP diets, thereby benefiting mucosal integrity, as also evidenced by an intestinal morphology similar to that of the PC diet. Notably, we observed no duodenal morphological differences, contrasting with previous reports [9,30,31], potentially due to segment-specific enzyme effects. Additionally, the catalytic power of exogenous proteases (neutral or alkaline) is directly determined by their pH-dependent activation, with neutral proteases exhibiting greater activity under the acidic-to-neutral conditions (characteristic of the duodenum and jejunum) [31].

The evaluation of the immune response evidenced that pro-inflammatory protein IL-6 expression was lower in the 1% CP-reduced diets compared with the 1.5% CP-reduced diets supplemented with protease B, suggesting that protease B may transiently stimulate immune activity, possibly through increased antigen exposure to poorly hydrolyzed anti-nutritional factors [32]. Similarly, regular CP diets with protease supplementation showed reduced IL-6 levels, indicating that proteases may mitigate inflammation by acting on anti-nutritional components. The observed IL-6 expressively activated by the treatment with the 1.5% CP-reduced diet supplemented with protease B may relate to acute inflammation caused by incomplete protease action on anti-nutritional factors, leading to increased haptoglobin production, as demonstrated in our results. This response correlates with elevated tumor necrosis factor-alpha (TNF-α), which regulates tight junction closure during gut inflammatory responses. Although we detected no significant changes in tight junction proteins (occludin and ZO-1), the intestinal inflammatory response observed primarily in the 1.5% CP-reduced diets with protease supplementation suggests mild subclinical inflammation. TNF-α plays a crucial role in systemic inflammation [33], while haptoglobin serves as a reliable biomarker of intestinal inflammation and stress [34]. These findings suggest that inappropriate CP reduction with protease supplementation may exacerbate post-weaning dysbiosis and inflammatory responses [35].

Protease supplementation, including animals fed the control diet without crude protein reduction, resulted in a lower incidence of diarrhea, as observed by Yu et al. [24] and Wang et al. [27]. This likely results from decreased undigested protein reaching the hindgut, thereby minimizing microbial fermentation and ammonia production [36,37]. However, these findings contradict the findings reported by Tactan et al. [38], possibly due to differences in diet composition, degree of CP reduction, and protease dosing. This effect suggests that the enzyme enhances protein digestibility and absorption in the gastrointestinal tract [9,31], thereby limiting the availability of substrates for undesirable microbial fermentation and consequently inhibiting the proliferation of pathogenic microorganisms associated with enteric disorders.

While proteases hydrolyze proteins into free amino acids and small peptides for improved intestinal absorption [39], our results demonstrated limited efficacy in compensating for amino acid deficiencies. Protease supplementation significantly increased the standardized ileal digestibility (SID) of methionine+cysteine (4.73%), tryptophan (7.37%), and cysteine (10.78%) but paradoxically reduced glycine (3.56%) and proline (8.64%) digestibility. This selective improvement aligns with Yu et al. [24] but contrasts with Wang et al. [27], who observed broader nutrient digestibility improvements. The reduced digestibility of glycine and proline may have exacerbated nutritional deficiencies in the 1.5% CP-reduced diets, contributing to the observed performance limitations. The reduction in digestibility of specific amino acids with protease supplementation is correlated with small, neutral amino acids (e.g., proline and glycine) and their absorption dynamics via oligopeptide transporters [5,32]. Rapid hydrolysis of these amino acids in the stomach and duodenum results in elevated concentrations of undigested free amino acids at the ileum. This occurs due to competitive inhibition at enterocyte oligopeptide transporters (PepT1), unlike methionine and threonine, which utilize distinct sodium-dependent transporters (e.g., B⁰AT1, ASC). Concurrently, protease supplementation promotes endogenous glycine secretion and enhances microbial utilization in the hindgut [5,40].

Additionally, is important to consider that protease efficacy depends on adequate substrate availability, as evidenced by the superior performance in regular CP diets versus CP-reduced diets; the 50 mg/kg protease dose in CP-reduced diets proved insufficient to overcome amino acid deficiencies; a uniform reduction of all amino acids in CP-reduced diets may create nutritional imbalances; and younger pigs (<20 kg) may benefit more from protease supplementation than heavier animals [41,42].

## 5. Conclusions

While protease supplementation can improve the digestibility of certain amino acids (Met+Cys and Thr), as well as protein digestibility, our findings suggest it cannot fully replace careful amino acid balancing in CP-reduced diets. However, protease-supplemented diets were associated with improved intestinal morphometry and a reduced incidence of diarrhea. Future research should investigate optimal protease dosing strategies and target amino acid reductions rather than uniform CP decreases.

## Figures and Tables

**Table 1:** Ingredient composition of experimental diets and calculated nutritional content for each feeding phase ^1^.

Feeding Phases	21–32 Days			32–42 Days			42–63 Days		
Ingredients (g/kg)	PC ^2^	NC1	NC1.5 ^3^	PC	NC1	NC1.5	PC	NC1	NC1.5
Corn, 7.88%	468	489	500	544	567	578	628	650	662
Micronized soybean	100	100	100	50	50	50	---	---	---
Soybean meal, 45%	170	148	137	240	218	207	300	278	267
Bovine plasma AP 920	40	40	40	20	20	20	---	---	---
Milk serum, powder	165	165	165	82	82	82	---	---	---
Soybean oil	10.0	10.0	10.0	22.7	22.3	21.9	28.9	28.3	28.0
Dicalcium phosphate	7.50	7.70	7.70	9.00	9.10	9.20	10.60	10.70	10.80
Limestone	6.60	6.70	6.80	7.00	7.10	7.20	9.60	9.70	9.70
Salt	3.00	3.00	3.00	4.50	4.50	4.50	4.90	4.90	4.90
Vitamin and mineral premix ^4^	3.40	3.40	3.40	3.00	3.00	3.00	2.40	2.40	2.40
Lysine-HCl	4.35	4.30	4.26	4.40	4.36	4.32	4.88	4.82	4.79
DL-Methionine	2.21	2.20	2.19	1.89	1.88	1.86	1.84	1.82	1.81
L-Threonine	2.23	2.18	2.15	2.18	2.13	2.10	2.07	2.01	1.99
L-Arginine	2.45	2.35	2.30	1.50	1.45	1.40	---	---	---
L-Isoleucine	0.20	0.15	0.13	---	---	---	---	---	---
L-Tryptophan	0.44	0.43	0.43	0.37	0.35	0.35	0.36	0.34	0.34
L-Valine	0.81	0.74	0.71	0.68	0.29	0.57	0.77	0.69	0.66
Zinc oxide	2.40	2.40	2.40	1.00	1.00	1.00	---	---	---
Choline chloride, 60%	0.88	0.88	0.88	0.82	0.82	0.82	0.76	0.76	0.76
Antioxidant	0.10	0.10	0.10	0.10	0.10	0.10	0.10	0.10	0.10
Phytase	0.05	0.05	0.05	0.05	0.05	0.05	0.05	0.05	0.05
Inert (Caulim)	10.23	11.04	11.45	5.00	5.00	5.00	4.60	4.60	4.60
Total	1000	1000	1000	1000	1000	1000	1000	1000	1000
Calculated nutritional value									
Metabolizable energy, kcal/kg	3400	3400	3400	3400	3400	3400	3350	3350	3350
Net energy, kcal/kg	2577	2589	2594	2607	2618	2623	2597	2607	2613
Crude protein, %	21.8	20.9	20.5	20.8	19.9	19.5	19.3	18.5	18.1
SID Lysine, %	1.45	1.39	1.36	1.35	1.29	1.26	1.25	1.20	1.17
SID Methionine, %	0.48	0.47	0.46	0.45	0.44	0.43	0.44	0.43	0.42
SID Met + Cis, %	0.81	0.79	0.78	0.75	0.73	0.72	0.71	0.69	0.68
SID Threonine, %	0.97	0.94	0.92	0.90	0.86	0.85	0.81	0.78	0.76
SID Tryptophan, %	0.28	0.26	0.26	0.26	0.24	0.24	0.24	0.23	0.22
SID Arginine, %	1.45	1.38	1.35	1.35	1.28	1.24	1.15	1.09	1.06
SID Valine, %	1.00	0.96	0.94	0.93	0.86	0.86	0.86	0.82	0.80
SID Isoleucine, %	0.80	0.76	0.74	0.76	0.72	0.70	0.71	0.68	0.66
SID Leucine, %	1.66	1.61	1.59	1.58	1.53	1.50	1.47	1.42	1.40
Total Ca, %	0.80	0.80	0.80	0.80	0.80	0.80	0.88	0.88	0.88
Available P, %	0.53	0.53	0.53	0.48	0.48	0.48	0.43	0.43	0.43
K, %	0.98	0.95	0.93	0.87	0.84	0.82	0.75	0.72	0.70
Na, %	0.35	0.35	0.35	0.30	0.30	0.30	0.21	0.21	0.21
Cl, %	0.50	0.50	0.50	0.47	0.47	0.47	0.36	0.36	0.36

^1^ PC: positive control diet; NC1: negative control diet with a 1.0% reduction in crude protein (CP) and amino acids; NC1.5: negative control diet with a 1.5% reduction in CP and amino acids. ^2^ PC+A: PC diet supplemented with 100 g/ton of commercial protease A. ^3^ NC1.5+A: NC1.5 diet supplemented with 50 g/ton of commercial protease A; NC1.5+B: NC1.5 diet supplemented with 50 g/ton of commercial protease B. ^4^ Per kg of complete diet: vitamin A, 7270.00 IU/kg; vitamin D3, 1,599,000.00 IU/kg; vitamin E, 43,600.00 IU/kg; vitamin K3, 3150.00 mg/kg; vitamin B1, 1066.00 mg/kg; vitamin B2, 3974.00 mg/kg; vitamin B6, 2133.00 mg/kg; vitamin B12, 22,000.00 mcg/kg; calcium D-pantothenate, 16.00 g/kg; niacin, 31.50 g/kg; biotin, 107.00 mg/kg; folic acid, 339.00 mg/kg; antioxidant additive BHT, 1000.00 mg/kg; Fe, 67.90 g/kg iron sulfate; Cu, 10.18 g/kg copper sulfate; Mn, 40.00 g/kg manganese oxide; Zn, 92.10 g/kg zinc oxide; I, 838.00 mg/kg calcium iodate; Se, 305.00 mg/kg sodium selenite.

**Table 2:** Sequence of primers used for gene expression analysis.

Gen	GenBank Code	Sequence
TNF-α	NM_214022.1	F:5′CATCGCCGTCTCCTACCA3′ R:5′CCCAGATTCAGCAAAGTCCA3′
IL-1β	NM_214055.1	F:5′TCTGCCCTGTACCCCAACTG3′ R:5′CCCAGGAAGACGGGCTTT3′
IL-6	NM_001252429.1	F:5′CCTGTCCACTGGGCACATAAC3′ R:5′CAAGAAACACCTGGCTCTGAAAC3′
Haptoglobin	NM_214000.2	F:5′GCTAAGAATCTCCGCTTGG3′ R:5′CAATCTCCACCTCCTGTTTC3′
IL-10	NM_214041.1	F:5′GAAGGACCAGATGGGCGACTT3′ R:5′CACCTCCTCCACGGCCCTTG3′
TGF-β1	NM_214015.1	F:5′GGACCTTATCCTGAATGCCTT3′ R:5′TAGGTTACCACTGAGCCACAAT3′
OCL	NM_001163647.1	F:5′TCCTGGGTGTGATGGTGTTC3′ R:5′CGTAGAGTCCAGTCACCGCA3′
ZO-1	XM_003353439.2	F:5′AAGCCCTAAGTTCAATCACAATCT 3R:5′ATCAAACTCAGGAGGCGGC3′
18S	AY_265350.1	F: GGCTACCACATCCAAGGAAG
		R: TCCAATGGATCCTCGCGGAA

TNF-α—tumor necrosis factor-alpha; IL-1β—interleukin 1 beta; IL-6—interleukin 6; IL-10—interleukin 10; TGF-β1—tumor necrosis factor beta 1; OCL—occluding; ZO-1—zonula occludens-1.

**Table 3:** Ingredient composition of experimental diets (as-fed basis).

Ingredients, %	Basal Diet	Basal Diet + Protease A	N-Free
Soybean meal	30	30	0
Soybean oil	5.00	5.00	4.00
Protease A ^1^	-	0.05	-
Sugar	20	20	20
Starch	36.58	36.53	67.58
Dicalcium phosphate	1.96	1.96	1.96
Limestone	0.76	0.76	0.76
Sodium chloride	0.40	0.40	0.40
Vitamin supplement ^2^	0.12	0.12	0.12
Minerals supplement ^3^	0.12	0.12	0.12
Potassium carbonate	0.40	0.40	0.40
Antioxidant	0.01	0.01	0.01
Magnesium oxide	0.10	0.10	0.10
Cellulose	4.00	4.00	4.00
Choline chloride	0.05	0.05	0.05
Titanium dioxide ^4^	0.50	0.50	0.50
Total	100.00	100.00	100.00

^1^ Production of bacterial synthesis by *Aspergillus* spp. and *Bacillus* spp. ^2^ Provided the following quantities per kilogram of complete diet: vitamin A, retinyl acetate, 7270.00 IU/kg; vitamin D3, cholecalciferol, 1,599,000.00 IU/kg; vitamin E, DL-alpha tocopherol, 43,600.00 IU/kg; vitamin K3, bisulfate of menadione nicotinamide, 3150.00 mg/kg; vitamin B1, thiamine mononitrate, 1066.00 mg/kg; vitamin B2, riboflavin, 3974.00 mg/kg; vitamin B6, pyridoxine hydrochloride, 2133.00 mg/kg; vitamin B12, 22,000.00 mcg/kg; calcium D-pantothenate, 16.00 g/kg; niacin, 31.50 g/kg; biotin, 107.00 mg/kg; folic acid, 339.00 mg/kg; antioxidant additive BHT, 1000.00 mg/kg. ^3^ Provided the following quantities per kilogram of complete diet: Fe, 67.90 g/kg iron sulfate; Cu, 10.18 g/kg; copper sulfate; Mn, 40.00 g/kg manganese oxide; Zn, 92.10 g/kg zinc oxide; I, 838.00 mg/kg calcium iodate; Se, 305.00 mg/kg sodium selenite. ^4^ Titanium dioxide (TiO_2_) is an indicator of indigestibility.

**Table 4:** Effects of protease supplementation in diets ^1^ with regular protein levels or reduced protein levels for piglets during the nursery phase.

Phase	PC	PC+A	NC1	NC1.5	NC1.5+A	NC1.5+B	SEM ^2^	*p*-Value
21 a 32								
ADG, g	0.223 a	0.225 a	0.213 ab	0.197 ab	0.188 b	0.189 b	0.005	0.038
ADFI, g/d	0.306	0.310	0.276	0.304	0.289	0.292	0.004	0.088
FCR	1.397 b	1.362 b	1.437 ab	1.544 ab	1.591 a	1.469 ab	0.022	0.005
32 a 42								
ADG, g	0.413 ab	0.431 a	0.415 ab	0.414 ab	0.385 b	0.392 b	0.005	0.043
ADFI, g/d	0.588	0.592	0.610	0.589	0.567	0.570	0.006	0.314
FCR	1.423 abc	1.359 c	1.391 abc	1.386 bc	1.460 ab	1.477 a	0.010	0.002
42 a 63								
ADG, g	0.586 a	0.580 a	0.578 ab	0.556 ab	0.527 b	0.542 ab	0.006	0.011
ADFI, g/d	0.929 a	0.906 ab	0.903 ab	0.916 ab	0.843 b	0.843 b	0.009	0.005
FCR	1.587 ab	1.534 b	1.583 ab	1.613 a	1.612 a	1.548 ab	0.007	0.003
21 a 63								
ADG, g	0.448 a	0.455 a	0.447 ab	0.430 ab	0.404 b	0.406 ab	0.005	0.004
ADFI, g	0.685 a	0.670 ab	0.673 ab	0.670 ab	0.635 ab	0.621 b	0.007	0.026
FCR	1.529 ab	1.484 b	1.510 ab	1.548 a	1.562 a	1.521 ab	0.007	0.006

a, b, c: Means in the rows followed by distinct letters differ by the Tukey test (*p* < 0.05). ^1^ PC: positive control diet; PC+A: PC diet supplemented with 100 g/ton of commercial protease A; NC1: negative control diet with a 1.0% reduction in crude protein (CP) and amino acids; NC1.5: negative control diet with a 1.5% reduction in CP and amino acids; NC1.5+A: NC1.5 diet supplemented with 50 g/ton of protease A; NC1.5+B: NC1.5 diet supplemented with 50 g/ton of protease B. ^2^ SEM, standard error of the mean.

**Table 5:** Effects of protease supplementation in diets ^1^ with regular protein levels or reduced protein levels on intestinal morphology of 32-day-old piglets.

Item	PC	PC+A	NC1	NC1.5	NC1.5+A	NC1.5+B SEM ^2^		*p*-Value
Duodenum								
Villus height, μm	344.68	358.29	363.75	379.00	331.71	358.95	4.91	0.221
Crypt depth, μm	401.64	384.35	381.39	384.51	390.18	386.54	5.19	0.905
VH:CD	0.88	0.94	0.97	0.99	0.87	0.93	0.02	0.490
Jejunum								
Villus height, μm	336.50	390.74	358.59	335.79	331.71	375.01	9.34	0.371
Crypt depth, μm	300.37 ab	248.46 b	289.01 ab	299.80 ab	305.00 a	283.13 ab	5.70	0.043
VH:CD	1.16 ab	1.61 a	1.26 ab	1.12 b	1.09 b	1.36 ab	0.05	0.017
Ileum								
Villus height, μm	380.02 a	376.33 ab	368.42 ab	338.91 ab	323.30 ab	308.50 b	7.41	0.014
Crypt depth, μm	241.94	221.87	243.78	242.20	223.35	234.44	4.96	0.656
VH:CD	1.62	1.73	1.52	1.40	1.48	1.36	0.04	0.964

a, b, Means in the row followed by distinct letters differ by the Tukey test (*p* < 0.05). ^1^ PC: positive control diet; PC+A: PC diet supplemented with 100 g/ton of commercial protease A; NC1: negative control diet with a 1.0% reduction in crude protein (CP) and amino acids; NC1.5: negative control diet with a 1.5% reduction in CP and amino acids; NC1.5+A: NC1.5 diet supplemented with 50 g/ton of protease A; NC1.5+B: NC1.5 diet supplemented with 50 g/ton of protease B. ^2^ SEM, standard error of the mean.

**Table 6:** Effects of protease supplementation in diets ^1^ with regular protein levels or reduced protein levels on gene expression of pro-inflammatory proteins in the jejunum of 32-day-old piglets.

Item ^2^	PC	PC+A	NC1	NC1.5	NC1.5+A	NC1.5+B	SEM ^3^	*p*-Value
IL-1β	4.074	3.597	5.057	4.244	5.073	4.883	0.183	0.0986
IL-6	1.935 ab	1.735 b	1.886 b	2.076 ab	2.452 ab	2.681 a	0.089	0.0061
IL-10	1.640	1.492	1.460	1.715	1.722	1.789	0.042	0.1381
TNF-α	2.068 b	2.018 b	2.586 ab	2.113 b	2.434 ab	2.843 a	0.073	0.0021
Haptoglobin	2.455 b	2.450 b	2.082 b	2.633 b	3.040 ab	3.611 a	0.103	0.0015
TGF-β1	2.164	2.082	2.408	2.157	2.361	2.506	0.055	0.1554
ZO-1	2.158	2.223	2.756	2.281	2.581	2.765	0.082	0.097
OCL	1.750	1.755	1.968	1.858	2.049	2.239	0.062	0.1341

a, b, Indicates that the means in the row followed by distinct letters differ by the Tukey test (*p* < 0.05). ^1^ PC: positive control diet; PC+A: PC diet supplemented with 100 g/ton of commercial protease A; NC1: negative control diet with a 1.0% reduction in crude protein (CP) and amino acids; NC1.5: negative control with a 1.5% reduction in CP and amino acids; NC1.5+A: NC1.5 diet supplemented with 50 g/ton of protease A; NC1.5+B: NC1.5 diet supplemented with 50 g/ton of protease B. ^2^ IL-1β—interleukin 1 beta; IL-6—interleukin 6; IL-10—interleukin 10; TNF-α—tumor necrosis factor-alpha; TGF-β1—tumor necrosis factor beta 1; OCL—occluding; ZO-1—zonula occludens-1. ^3^ SEM, standard error of the mean.

**Table 7:** Standardized ileal digestibility coefficients (SID) of crude protein and amino acids from basal diet and basal diet with protease A (dry matter).

Item	Basal Diet	Basal Diet + Protease A	SEM ^1^	*p*-Value
Crude protein, %	83.45	82.04	0.5931	0.2480
Essential AA				
Arginine, %	93.43	93.72	0.2674	0.6120
Phenylalanine, %	88.02	87.61	0.3568	0.5850
Histidine, %	89.41	88.45	0.3928	0.2380
Isoleucine, %	86.25	86.15	0.4429	0.9210
Leucine, %	87.07	86.36	0.4085	0.4080
Lysine, %	89.63	89.21	0.4741	0.6730
Methionine, %	95.36	94.60	0.3643	0.3180
Met + Cys, %	89.79 b	94.52 a	0.7544	0.0001
Threonine, %	82.54	81.09	0.7106	0.3240
Tryptophan, %	74.53 b	81.90 a	1.3195	0.0011
Valine, %	83.63	82.64	0.5391	0.3800
Non-essential AA				
Aspartic Acid, %	85.31	84.49	0.3930	0.3210
Glutamic Acid, %	88.43	88.02	0.3054	0.5200
Alanine, %	80.48	79.94	0.7315	0.7270
Cysteine, %	82.74 b	93.52 a	1.5490	<0.0001
Glycine, %	81.50 a	77.98 b	0.8682	0.0367
Proline, %	90.97 a	82.33 b	1.6975	0.0048
Serine, %	86.27	84.14	0.7002	0.1340
Tyrosine, %	85.95	84.25	0.4899	0.0816

a, b: Means in the row followed by distinct letters differ by the F Test (*p* < 0.05). ^1^ SEM, standard error of the mean.

## Data Availability

Data are contained within the article and Supplementary Materials.

## Supplementary Materials

The following supporting information can be downloaded at: https://www.mdpi.com/article/10.3390/ani15142109/s1, Table S1: Nutritional composition of the experimental diets for each feeding phase (in as-is basis) and Table S2: Apparent ileal digestibility coefficients (AID) of CP and of AAs from basal diet and basal diet + protease A (dry matter).
