# Supplementary material for: Protease Enzyme Supplementation in Weaning Piglets Fed Reduced Crude Protein Diets: Effects on Gut Health Integrity and Performance Response
Source: Animals (Basel). 2025 Jul 17;15(14):2109. doi: 10.3390/ani15142109 (PMC12291861; doi:10.3390/ani15142109)
Supplement: Supplementary file 1 [file animals-15-02109-s001.zip › animals-3720744-supplementary.pdf]

Supplementary Materials

Table S1. Nutritional composition analyzed of the experimental diets[1] for each feeding phase (in as is basis).

**Feeding phases**	**21-32 d**						**32-42 d**						**42-63 d**					
**Nutrients, %**	**PC**	**PC+A**	**NC1**	**NC1.5**	**NC1.5+A**	**NC1.5+B**	**PC**	**PC+A**	**NC1**	**NC1.5**	**NC1.5+A**	**NC1.5+B**	**PC**	**PC+A**	**NC1**	**NC1.5**	**NC1.5+A**	**NC1.5+B**
Dry matter	90.3	90.3	90.1	90.1	90.6	90.5	89.7	89.7	89.5	89.3	89.2	89.7	89.2	89.2	89.6	89.7	89.3	89.6
Aspartic acid	2.10	2.12	2.06	1.98	2.08	2.00	2.07	2.05	2.04	1.90	2.25	2.02	2.07	2.17	2.15	1.78	2.06	1.95
Glutamic acid	3.58	3.51	3.41	3.28	3.25	3.18	3.33	3.49	3.23	3.23	3.44	3.14	3.41	3.37	3.26	3.04	3.12	3.30
Alanine	1.04	1.05	1.03	0.97	1.02	0.99	0.99	1.00	0.97	0.94	1.03	0.95	1.00	0.99	0.95	0.88	0.94	0.96
Arginine	1.51	1.50	1.41	1.29	1.35	1.30	1.35	1.40	1.28	1.24	1.36	1.25	1.28	1.27	1.22	1.09	1.15	1.22
Cystine	0.17	0.19	0.18	0.15	0.18	0.17	0.17	0.17	0.16	0.14	0.16	0.15	0.15	0.16	0.14	0.13	0.13	0.15
Phenylalanine	0.99	0.98	0.95	0.90	0.91	0.88	0.92	0.95	0.89	0.88	0.94	0.86	0.94	0.93	0.88	0.82	0.84	0.89
Glycine	0.81	0.81	0.77	0.75	0.74	0.72	0.76	0.79	0.73	0.73	0.78	0.72	0.79	0.78	0.75	0.70	0.72	0.75
Hydroxyproline	0.02	0.02	0.02	0.02	0.02	0.02	0.02	0.02	0.02	0.02	0.02	0.02	0.03	0.03	0.03	0.03	0.03	0.03
Histidine	0.56	0.61	0.58	0.55	0.56	0.56	0.57	0.56	0.56	0.54	0.57	0.54	0.52	0.55	0.51	0.47	0.53	0.52
Isoleucine	0.96	0.94	0.92	0.89	0.87	0.86	0.85	0.91	0.84	0.82	0.89	0.79	0.88	0.85	0.77	0.73	0.73	0.79
Leucine	1.90	1.90	1.86	1.82	1.81	1.76	1.78	1.80	1.75	1.75	1.83	1.70	1.78	1.73	1.69	1.60	1.65	1.72
Lysine	1.47	1.68	1.46	1.45	1.46	1.46	1.34	1.43	1.27	1.31	1.37	1.32	1.30	1.28	1.32	1.22	1.27	1.29
Methionine	0.42	0.46	0.42	0.43	0.42	0.42	0.41	0.44	0.40	0.38	0.39	0.38	0.40	0.38	0.38	0.38	0.38	0.37
Proline	1.26	1.25	1.22	1.20	1.19	1.15	1.19	1.22	1.17	1.18	1.22	1.15	1.19	1.18	1.10	1.05	1.10	1.13
Serine	1.08	1.11	1.04	1.01	1.02	0.97	1.03	1.01	0.96	0.98	1.03	0.97	0.99	0.98	1.00	0.92	0.96	1.00
Taurine	ND	ND	ND	ND	ND	ND	ND	ND	ND	ND	ND	ND	ND	ND	ND	ND	ND	ND
Tyrosine	0.74	0.73	0.70	0.69	0.70	0.67	0.67	0.70	0.64	0.63	0.67	0.63	0.70	0.68	0.64	0.61	0.62	0.67
Threonine	1.06	1.13	1.06	1.03	1.06	1.01	1.00	1.00	0.91	0.90	0.96	0.91	0.87	0.89	0.89	0.83	0.85	0.86
Tryptophan	0.35	0.31	0.32	0.32	0.33	0.33	0.29	0.33	0.26	0.27	0.27	0.27	0.28	0.24	0.26	0.24	0.29	0.27
Valine	1.12	1.12	1.07	1.04	1.04	1.01	0.98	1.04	0.95	0.93	0.97	0.91	0.96	0.95	0.87	0.81	0.82	0.87
Total amino acids	21.2	21.4	20.5	19.8	20.0	19.5	19.7	20.3	19.0	18.8	20.2	18.7	19.5	19.4	18.8	17.3	18.2	18.8
Crude protein	22.1	21.9	21.3	20.4	20.6	20.8	20.9	21.2	19.6	20.1	20.2	19.6	20.2	19.6	19.8	18.6	18.9	19.3
Total nitrogen	3.54	3.50	3.41	3.26	3.30	3.32	3.34	3.39	3.14	3.22	3.23	3.14	3.23	3.14	3.17	2.98	3.02	3.08

[1]PC: Control diet; PC+A: PC diet supplemented with 100 g/ton of commercial protease A; NC1: Control with a 1.0% reduction in crude protein (CP) and amino acids; NC1.5: negative control with a 1.5% reduction in CP and amino acids; NC1.5+A: NC1.5 diet supplemented with 50 g/ton of protease A; NC1.5+B: NC1.5 diet supplemented with 50 g/ton of protease B. ND = not detected.

Table S2. Apparent ileal digestibility coefficients (AID) of CP and AAs from Basal diet and Basal diet+protease A (dry matter).

**Item**	**Basal diet**	**Basal diet+protease A**	**SEM**[1]	***p*-value**
Crude protein, %	75.47	73.89	0.6005	0.2000
Indispensable AA				
Arginine, %	90.09	90.35	0.2668	0.6510
Phenylalanine, %	84.20	83.83	0.3559	0.6280
Histidine, %	85.49	84.44	0.3969	0.1990
Isoleucine, %	81.74	81.71	0.4427	0.9750
Leucine, %	82.66	81.91	0.4096	0.3850
Lysine, %	86.20	85.78	0.4741	0.6730
Methionine, %	88.93	87.82	0.3802	0.1500
Met + Cys, %	82.61b	87.12a	0.7303	0.0002
Threonine, %	74.96	73.39	0.7150	0.2890
Tryptophan, %	62.81b	73.34a	1.6814	<0.0001
Valine, %	77.54	76.55	0.5391	0.3800
Dispensable AA				
Aspartic Acid, %	82.94	82.07	0.3954	0.2910
Glutamic Acid, %	85.86	85.42	0.3062	0.4940
Alanine, %	72.66	72.12	0.7315	0.7280
Cysteine, %	74.61b	85.38a	1.5490	<0.0001
Glycine, %	68.59a	64.89b	0.8844	0.0292
Proline, %	76.29a	67.46b	1.7156	0.0042
Serine, %	80.09	77.74	0.7128	0.1010
Tyrosine, %	81.12	79.42	0.4899	0.0816

[a, b] indicate significant differences by the F Test ($p<0.05$).

[1]SEM, standard error of the mean.

Baseline endogenous losses were determined by the Nitrogen -free diet (g/kg of daily DM intake): Lysine, 0.340; Methionine, 0.122; Threonine, 0.500; Tryptophan, 0.223; Arginine, 0.364; Valine, 0.426; Isoleucine, 0.311; Leucine, 0.555; Histidine, 0.172; Phenylalanine, 0.314; Alanine, 0.524; Cysteine, 0.122; Tyrosine, 0.232; Glycine, 0.890; Serine, 0.531; Proline, 1.189; Glutamic Acid, 0.740; Aspartic Acid, 0.416.
